# Comparison of radial immunodiffusion, turbidimetric immunoassay, and Brix refractometry for determining bovine colostrum quality

**DOI:** 10.3168/jdsc.2024-0604

**Published:** 2024-07-14

**Authors:** T.A. Westhoff, E.L. Behling-Kelly, S. Mann

**Affiliations:** Department of Population Medicine and Diagnostic Sciences, College of Veterinary Medicine, Cornell University, Ithaca, NY 14853

## Abstract

Determining the concentration of IgG in colostrum is critical for assessment of colostrum quality. On-farm use of a Brix refractometer to estimate colostrum IgG concentration is widespread, whereas radial immunodiffusion (RID) is the laboratory reference method. Turbidimetric immunoassay (TIA) might offer an alternative method to quantify IgG in colostrum, but the agreement with RID, as well as critical thresholds to determine high-quality colostrum, remain uncertain. The objective of this study was to determine the level of agreement between RID, Brix %, and TIA for evaluation of colostrum quality. Composite colostrum samples (n = 58) from Holstein cows were evaluated using a digital Brix refractometer at the time of collection and stored at −20°C until analysis. The concentration of IgG was determined using RID and TIA. Data were analyzed using Passing-Bablok regression and Bland-Altman plots. Critical thresholds for TIA and Brix measurements to identify colostrum with an IgG concentration ≥50 and ≥100 g/L based on the reference method were determined using logistic regression and receiver operating characteristic curves. Results revealed that both TIA (Kendall's τ = 0.91) and Brix % (τ = 0.78) had a strong correlation with RID. Passing-Bablok regression identified a systematic (6.91 [4.33 to 8.98] g/L) and proportional (0.69 [0.67 to 0.72] g/L) bias between RID and TIA. The optimum thresholds to identify samples with an IgG concentration ≥50 and ≥100 g/L were 40.6 g/L (area under the curve [AUC]: 1.0; sensitivity (Se): 100; specificity [Sp]: 100) and 85.8 g/L (AUC: 0.99; Se: 96.6; Sp: 96.6) for TIA and 18.4% (AUC: 1.0; Se: 100; Sp: 100) and 25.8% (AUC: 0.99; Se: 82.8; Sp: 93.1) for Brix %, respectively. Using the identified thresholds, our results show that both Brix and TIA were highly accurate for identifying high-quality colostrum, but because of a proportional bias, direct comparison of IgG concentration results obtained by RID and TIA are cautioned.

## Highlights

• Systematic and proportional bias was observed for TIA compared with RID.
• Brix % showed a strong correlation with RID.
• TIA cutpoints of 40.6 and 85.8 g/L accurately predicted RID IgG ≥50 and ≥100 g/L.
• Brix cutpoints of 18.4% and 25.8% accurately predicted RID IgG ≥50 and ≥100 g/L.

## Graphical Abstract

**Figure:**
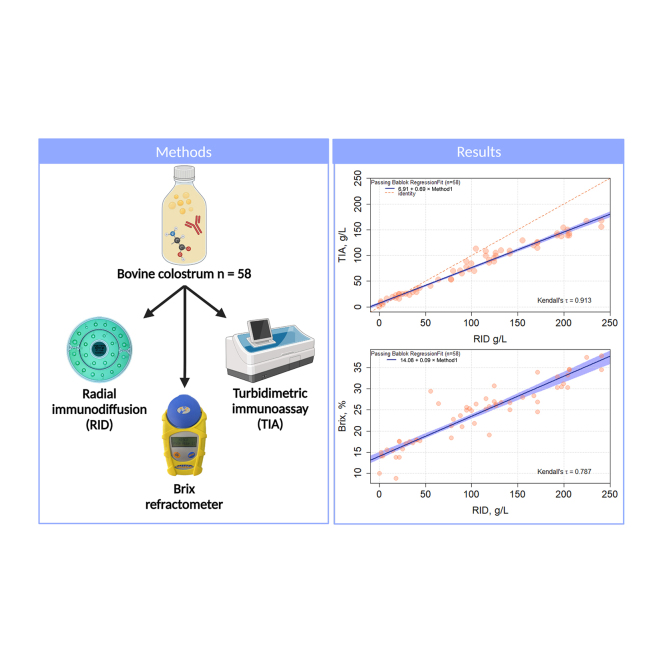
**Summary:** This study evaluated the agreement of turbidimetric immunoassay (TIA) and Brix % with the reference method, radial immunodiffusion (RID), for evaluation of colostrum quality. The results show a strong correlation between RID and TIA as well as Brix %. However, a systematic and proportional bias was observed for TIA, which indicates caution should be used for the direct comparison of immunoglobulin G (IgG) results determined via RID.

Neonatal calves acquire passive immunity via absorption of Ig from colostrum (Lopez and Heinrichs, 2022). Although colostrum contains low concentrations of IgM and IgA, IgG comprises 85% to 90% of Ig in colostrum (Larson et al., 1980). Thus, the concentration of IgG has historically been used to define colostrum quality, with high-quality colostrum containing ≥50 g of IgG/L (Godden et al., 2019). Radial immunodiffusion (**RID**) is the current laboratory-based reference method for direct quantification of IgG (Bartens et al., 2016; Ahmann et al., 2021). Because RID is cost- and time-prohibitive for on-farm analysis, use of a Brix refractometer provides a rapid and affordable indirect assessment of colostrum quality for on-farm use that has shown a strong correlation (r = 0.64 to 0.75) with the concentration of IgG determined by RID (Bielmann et al., 2010; Quigley et al., 2013; Bartier et al., 2015; Morrill et al., 2015; Röder et al., 2023).

Based on the principle of antigen-antibody complex scattering light, turbidimetric immunoassay (**TIA**) was originally described to quantify IgG in serum for assessment of transfer of passive immunity and has shown a positive correlation with results obtained by RID (r = 0.77 to 0.99; Etzel et al., 1997; Davis et al., 2005; Alley et al., 2012). In recent years, the use of TIA has been extended to determine the concentration of IgG in colostrum (Quigley et al., 2013; Breuer et al., 2023; Kerwin et al., 2023). However, when a laboratory and point-of-care TIA were used to determine IgG concentration in colostrum, systematic and proportional bias were observed such that TIA resulted in lower results for IgG concentration compared with RID (Quigley et al., 2013; Breuer et al., 2023). As such, the utility of TIA to quantify colostral IgG concentration remains uncertain and our understanding of the agreement between TIA and RID is hampered by limited validation work. Therefore, the objective of this study was to determine the level of agreement between RID, Brix %, and TIA for evaluation of colostrum quality.

All study procedures were approved by the Cornell University Institutional Animal Care and Use Committee (protocol numbers 2019–0031 and 2022–0167). Composite colostrum samples from Holstein cows (n = 112) were collected at first milking on 2 dairy farms in New York State. Brix % was determined using a digital Brix refractometer (model PA201, Misco) that was zero-set and calibrated according to the manufacturer's instructions. The resulting Brix % were used to select a convenience sample of colostrum samples (n = 58) to cover a spectrum of expected IgG concentration (Jensen and Kjelgaard-Hansen, 2006). The median (quartile 1, quartile 3) [range] Brix was 25.1% (17.6%, 28.6%) [8.9% to 37.8%]. Cows were entering parities 1 (n = 11; 19.0%), 2 (n = 10; 17.2%), 3 (n = 19; 32.8%), 4 (n = 12; 20.7%), 5 (n = 5; 8.6%), and 6 (n = 1; 1.7%). Colostrum was subsequently frozen at −20°C for IgG analysis by RID and TIA. Radial immunodiffusion was performed according to the manufacturer's instructions (Triple J Farms). Briefly, whole colostrum was thawed and warmed to room temperature, vigorously vortexed, and diluted 8-fold with sterile saline warmed to 37°C. Five microliters of each diluted unknown sample as well as the pooled bovine reference sera (28, 14, and 2.8 g/L) and a control sample were added to RID plates containing anti-bovine IgG in an agarose gel containing 0.1 *M* phosphate buffer pH 7.0, 0.1% sodium azide, 1 µg/mL amphotericin B, and 0.002 *M* EDTA. End-point precipitin ring diameter was measured after a 24-h incubation at room temperature using a 10× scale loupe as previously described (Mann et al., 2020). The squared diameters of the reference samples were plotted against the IgG concentration to create a linear standard curve. The inter-assay coefficient of variation determined on 3 plates was 4.7%.

Turbidimetric immunoassay was performed by the Clinical Pathology Laboratory at the Animal Health and Diagnostic Center (Cornell University). Colostrum was centrifuged at 4,000 × *g* for 30 min at 4°C to remove the lipid layer, diluted 8-fold with sterile saline, and the concentration of IgG was determined using a commercially available assay for bovine IgG (Midland BioProducts Corp.) on a Roche Cobas 6000 c501 analyzer (Roche Diagnostics Corp.) according to the methods described in Etzel et al. (1997). The assay had a linear range of detection of 0 to 32 g/L and was supplied with calibrators to generate a 6-point standard curve. The intra- and inter-assay CV were 1.7% and 1.9%, respectively.

The sample size was selected to achieve a spectrum of expected IgG concentration and surpass the minimum recommendation of 40 samples (Jensen and Kjelgaard-Hansen, 2006). Nonparametric Passing-Bablok regressions and Kendall rank correlation coefficients were generated in JMP Pro (v. 17.0.0; SAS Institute Inc.). The intercept of the Passing-Bablok regression can be interpreted as the systematic bias, and the slope measures the proportional bias between 2 methods. Methods are considered comparable when the 95% CI for the intercept include 0 and the slope includes 1 (Jensen and Kjelgaard-Hansen, 2006). A Bland-Altman plot was created in Graphpad (v. 9.5.1; GraphPad Software LLC) to visualize the mean and 95% CI agreement between methods. The concentrations of IgG determined by TIA as well as Brix % were evaluated for the optimum threshold associated with RID cut-points (≥50 and ≥100 g/L) using binary logistic regression models in JMP Pro (version 17.0.0). A receiver operating characteristic curve was used to identify the optimum threshold defined by the point with the highest combined sensitivity and specificity. Area under the curve (**AUC**) 0.70 ≤ AUC < 0.90 was considered accurate and AUC ≥0.90 was considered highly accurate (Swets, 1988). Sensitivity and specificity with 95% CI at the defined threshold were determined using MedCalc Statistical Software (MedCalc Software Ltd., version 23.0.2). Sensitivity was defined as the probability that TIA or Brix correctly classified samples as ≥50 or ≥100 g/L. Specificity was defined as the probability TIA or Brix correctly classified samples as <50 or <100 g/L.

The median ± SD (range) was 101.3 ± 70.9 (0 to 240) g/L for RID, 84.8 ± 49.2 (0.1 to 168) g/L for TIA, and 25.1 ± 6.8% (8.9% to 37.8%) Brix. Out of the 58 samples, 40 (69.0%) and 29 (50.0%) samples had an IgG concentration of ≥50 and ≥100 g/L when measured by RID, respectively. A strong correlation was observed between RID and TIA (τ = 0.91) as well as Brix % (τ = 0.78; Figure 1). However, the regression analysis identified a systematic and proportional bias for TIA. Turbidimetric immunoassay had an intercept (95% CI) of 6.91 (4.33 to 8.98) g/L and a proportional bias such that TIA resulted in an increase of 0.69 (0.67 to 0.72) g/L for every 1 g/L increase in IgG determined by RID. The Bland-Altman plot revealed a mean (95% CI) bias for TIA of −24.55 (−71.27 to 22.17) g/L compared with RID (Figure 2), but the interpretation of the mean bias alone must be done with caution in recognition of the proportional bias described above.

**Figure 1:**
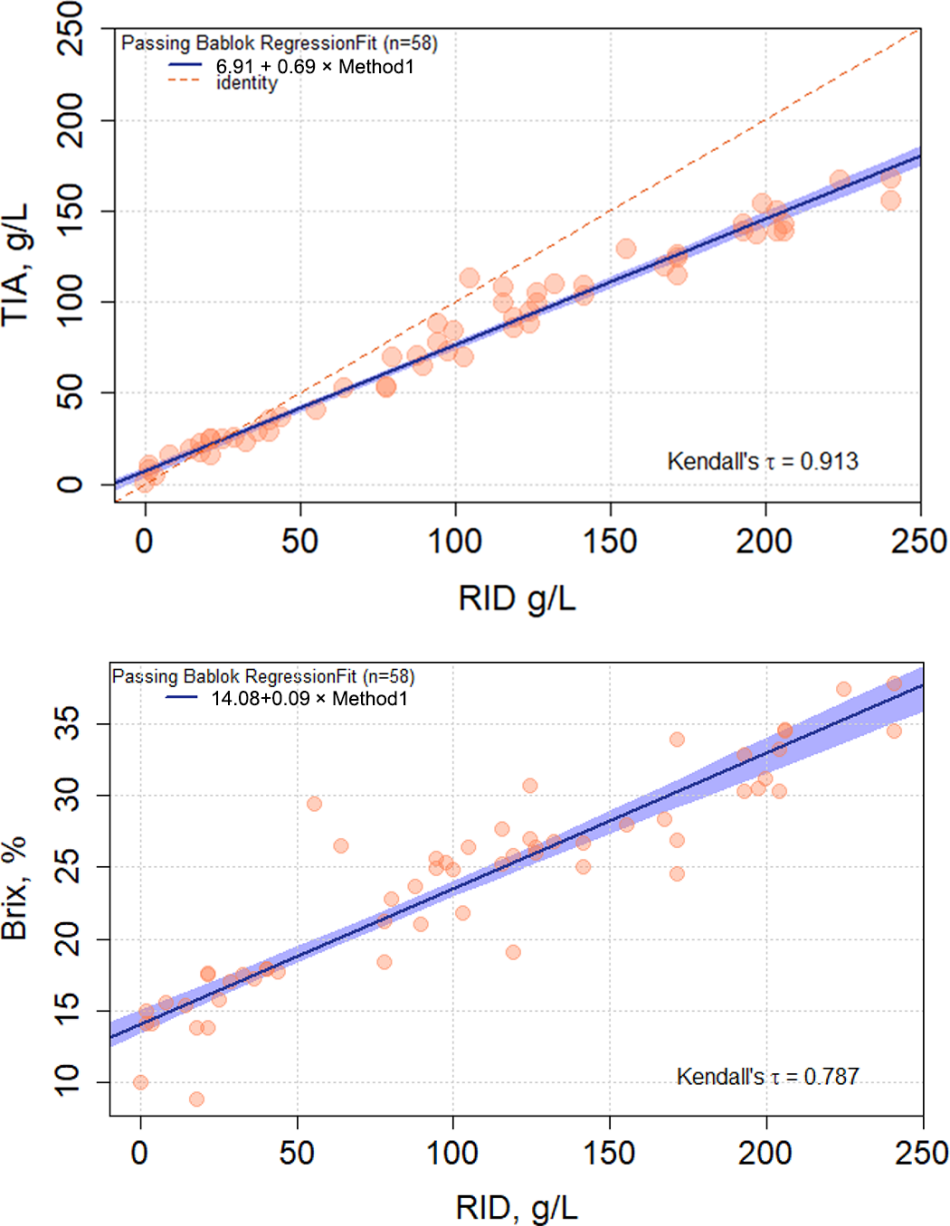
Passing-Bablok regressions for turbidimetric immunoassay (TIA) and a digital refractometer compared with radial immunodiffusion (RID). The blue line represents the regression line with 95% confidence intervals. The red dashed line represents the line of identity when the results from each method have the same unit.

**Figure 2:**
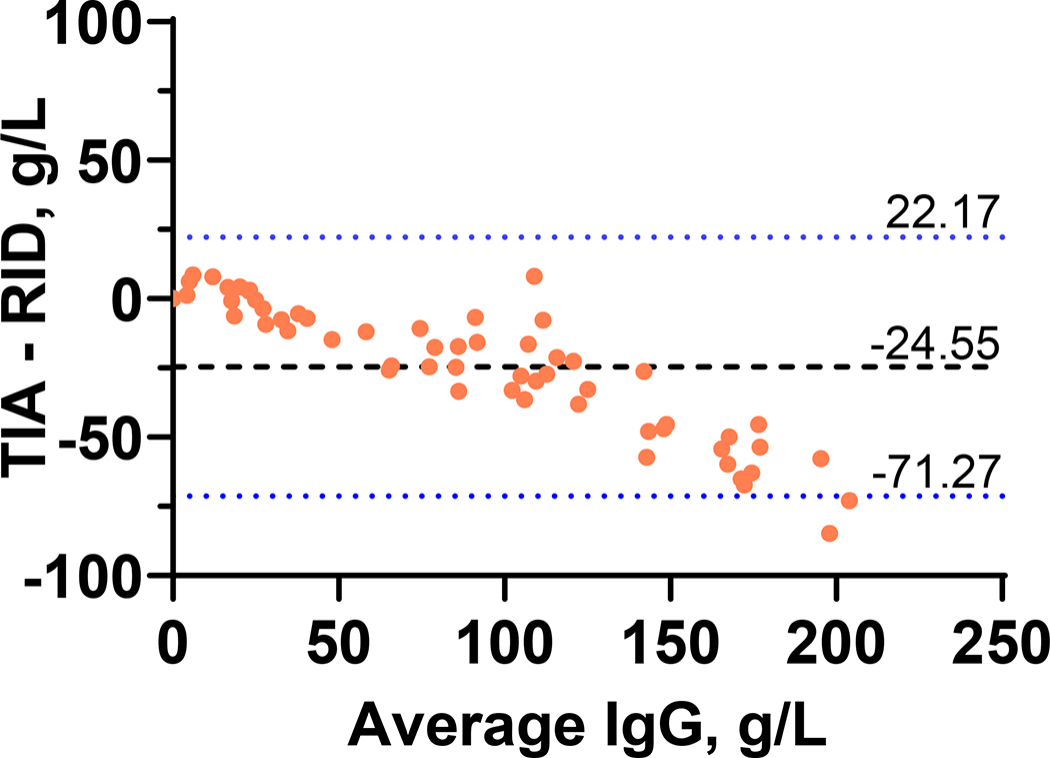
Bland-Altman plot for the difference in IgG concentration in colostrum samples (n = 58) determined using turbidimetric immunoassay (TIA) and radial immunodiffusion (RID). The dashed black line represents the mean bias, and the dotted blue lines represent the 95% CI agreement between methods.

Test characteristics to identify colostrum with ≥50 and ≥100 g of IgG/L are shown in Table 1. Turbidimetric immunoassay was highly accurate for the prediction of samples with RID IgG ≥50 and ≥100 g/L using the thresholds of 40.6 g/L (AUC: 1.0; Se: 100% [91.2% to 100%]; Sp: 100% [81.5% to 100%]) and 85.8 g/L (AUC: 0.99; Se: 96.6% [82.2% to 99.9%]; Sp: 96.6% [82.2% to 99.9%]), respectively. For the Brix refractometer, 18.4% (AUC: 1.0; Se: 100% [91.2% to 100%]; Sp: 100% [81.5% to 100%]) and 25.8% (AUC: 0.94; Se: 82.8% [64.2% to 94.2%]; Sp: 93.1% [77.2% to 99.2%]) were identified as the optimum threshold for the prediction of IgG ≥50 and ≥100 g/L, respectively.

**Table 1:** Test characteristics for turbidimetric immunoassay (TIA) and a Brix refractometer to determine colostrum with an IgG concentration ≥50 and ≥100 g/L determined by the reference method radial immunodiffusion

Method	Cut-point, g/L	Threshold^1^	AUC^2^	Se^3^ (95% CI)	Sp^4^ (95% CI)
TIA, g/L	≥50	40.6	1.0	100	100
				(91.2 to 100)	(81.5 to 100)
	≥100	85.8	0.99	96.6	96.6
				(82.2 to 99.9)	(82.2 to 99.9)
Brix,^5^ %	≥50	18.4	1.0	100	100
				(91.2 to 100)	81.5 to 100)
	≥100	25.8	0.94	82.8	93.1
				(64.2 to 94.2)	(77.2 to 99.2)

1 Defined by the point with the highest combined sensitivity and specificity.

2 Area under the curve.

3 Sensitivity was defined as the proportion of samples identified as ≥50 or ≥100 g/L using the tested method.

4 Specificity was defined as the proportion of samples identified as <50 or <100 g/L using the tested method.

5 Model PA201, Misco.

Although a strong correlation was observed, our results identified a systematic and proportional bias between TIA and RID. The lack of agreement between RID and TIA, specifically at higher concentrations of Ig, was previously demonstrated in bovine colostrum and serum (Quigley et al., 2013; Breuer et al., 2023; Kreuder et al., 2023), foal plasma (Ujvari et al., 2017), as well as in plasma from humans (Bergström and Lefvert, 1980). Kinetics of antibody-antigen reactions are complex and can be affected by the epitope and affinity of the antibody as well as the ratio of antigen to antibody that can affect the equivalence point (Price and Spencer, 1981; Price et al., 1983). Selective transport of IgG_1_ into the mammary gland during colostrogenesis results in a disproportionate concentration of IgG_1_ and IgG_2_ in colostrum compared with in circulation. Since the TIA range of detection was determined using serum, the affinity of IgG subclasses for specific epitopes could have resulted in samples with higher concentrations of IgG to reach near equivalence, diminishing the rate or stability of antigen-antibody complex. Including polyethylene glycol in assay reagents has been used as a method to displace the equivalence point and increase precipitin reaction, and assay sensitivity and stability (Van Munster et al., 1977; Bergström and Lefvert, 1980). In Bergström and Lefvert (1980) and Byrjalsen and Ingwersen (1985), use of polyethylene glycol in assay reagents at 9% or 10% increased assay sensitivity by 10% and reduced sample blank interference, respectively, when determining constituents of serum or plasma compared with the use of assay reagents with lower and higher concentrations of polyethylene glycol. Although polyethylene glycol was included in the assay buffer at 5% concentration in the current study, the assay sensitivity and stability when using lower and higher concentration of polyethylene glycol should be considered.

Colostrum also contains a diverse profile of sugars, proteins, minerals, and bioactive factors that could create a matrix effect by influencing the antigen-antibody reaction or light scattering properties. In Price and Spencer (1981), the turbidimetric reaction was affected by the concentration of Ca, Mg, and Cu. Moreover, when using an ELISA to determine the concentration of IgG in colostrum, extensive dilution (1 × 10^5^ to 1.6 × 10^6^) was required to overcome the matrix effect of colostrum (Baumrucker et al., 2014). Thus, it is plausible that the 8-fold dilution in the current study needed to get the predicted IgG concentration into the mid-standard range was not sufficient to overcome the matrix effect from the complex mixture of components in colostrum that could interfere with TIA performance.

Even though the RID kit used in the current study has exhibited minimal plate and lot variability (Thompson et al., 2023), the accuracy and precision of results are dependent on the provided standards and the ability and accuracy of the user in determining the precipitant diameter, as well as in pipetting small volumes, and the dilution factor. Poor agreement has been reported between results obtained from commercially available RID kits (Ameri and Wilkerson, 2008) as well as for the IgG concentration of commercial standard solutions (Breuer et al., 2023) suggesting that, albeit the reference method, imprecision exists within RID.

Given the bias between RID and TIA, direct comparison of IgG results obtained from these methods is not warranted. However, TIA was highly accurate in correctly distinguishing samples with ≥50 and ≥100 g of IgG/L determined using RID. Using a point-of-care TIA, the cut-points to identify colostrum with an RID IgG concentration <50 and <100 g/L were 28.5 g/L (AUC: 0.97) and 38.8 g/L (AUC: 0.88) for fat-separated colostrum, respectively (Breuer et al., 2023) and were lower than the thresholds identified in the current study. While differences in TIA methods between studies should be acknowledged, small sample sizes (n = 58 and n = 206) as well as the prevalence of samples with <50 g/L (n = 18 [31.0%] and n = 16 [7.8%]) and <100 g/L (n = 29 [50.0%] and n = 62 [30.1%]) were included in the current and aforementioned study, respectively.

The strong correlation between RID and Brix reported in the current study was consistent with correlations reported previously (r = 0.64 to 0.75; Bielmann et al., 2010; Quigley et al., 2013; Bartier et al., 2015; Morrill et al., 2015; Röder et al., 2023). Authors have identified cut-points for the prediction of samples with ≥50 and ≥100 g of IgG/L between 21.0% and 23.0% (Bielmann et al., 2010; Quigley et al., 2013; Bartier et al., 2015) and 23.8% and 23.9% (Breuer et al., 2023; Mann et al., 2024), respectively. A meta-analysis by Buczinski and Vandeweerd (2016) revealed that a threshold of ≥22.0% should be used to diagnose samples with an IgG concentration ≥50 g/L, whereas <18.0% should be used to identify poor quality samples.

It is worth noting that using Youden's J statistic in individual studies with small sample sizes can result in bias of determined cut-points and associated Se and Sp estimates compared with the optimum population cut-point and accuracy measurements (Leeflang et al., 2008; Bhandari et al., 2021). In the future, additional primary studies, with sufficient sample sizes, are needed comparing TIA and RID to identify optimal cut-points in colostrum through meta-analyses to prevent this bias.

Our results show that IgG concentration determined by RID strongly correlates with TIA and Brix. Turbidimetric immunoassay and the Brix refractometer were highly accurate in identifying samples with ≥50 and ≥100 g/L in the current study, but additional studies are needed to identify the optimum cut-points using TIA at the population level. Because a systematic and proportional bias was observed for TIA when compared with RID, the direct comparison of results between methods is not recommended.
